# Going circular: history, present, and future of circRNAs in cancer

**DOI:** 10.1038/s41388-023-02780-w

**Published:** 2023-08-16

**Authors:** Giuseppina Pisignano, David C. Michael, Tanvi H. Visal, Radu Pirlog, Michael Ladomery, George A. Calin

**Affiliations:** 1 Department of Life Sciences, University of Bath, Claverton Down, Bath, BA2 7AY UK; 2 Department of Experimental Therapeutics, The University of Texas MD Anderson Cancer Center, Houston, TX USA; 3 Center for RNA Interference and Non-Coding RNAs, The University of Texas MD Anderson Cancer Center, Houston, TX USA; 4 Faculty of Health and Applied Sciences, University of the West of England, Coldharbour Lane, Frenchay, Bristol, BS16 1QY UK

**Keywords:** Cancer genetics, Diagnostic markers

## Abstract

To date, thousands of highly abundant and conserved single-stranded RNA molecules shaped into ring structures (circRNAs) have been identified. CircRNAs are multifunctional molecules that have been shown to regulate gene expression transcriptionally and post-transcriptionally and exhibit distinct tissue- and development-specific expression patterns associated with a variety of normal and disease conditions, including cancer pathogenesis. Over the past years, due to their intrinsic stability and resistance to ribonucleases, particular attention has been drawn to their use as reliable diagnostic and prognostic biomarkers in cancer diagnosis, treatment, and prevention. However, there are some critical caveats to their utility in the clinic. Their circular shape limits their annotation and a complete functional elucidation is lacking. This makes their detection and biomedical application still challenging. Herein, we review the current knowledge of circRNA biogenesis and function, and of their involvement in tumorigenesis and potential utility in cancer-targeted therapy.

## Background: the advent of circRNAs

Despite their recent fame, the discovery of circRNAs dates back over 40 years. CircRNAs were first discovered in the murine respirovirus (*Sendai virus*) [1] and in plant pathogenic viruses termed viroids [2]. The initial physical evidence of the existence of a circular form of RNA was obtained by electron microscope analysis of the cytoplasmic fraction of eukaryotic (HeLa) cells in 1979 [3] and later in 1986 when they were identified within the hepatitis delta virus (HDV) [4]. Such circular shaped viral genomes possess the distinct property of generating multiple copies of an RNA through the rolling circle replication mechanism that facilitates the spread of infection more efficiently. However, evidence of another type of circRNA as intermediate molecules excised from pre-mRNA started emerging [5–7]. In 1991, a group of researchers found the first examples of endogenous spliced circRNAs in humans, transcribed from the *DCC* gene and end-joined in a scrambled order compared to the canonical linear sequence [8]. In parallel, the mouse *Sry* gene, encoding a crucial molecule responsible for sex determination during embryogenesis, was found to be expressed in adult mice testes exclusively as a 1.23-kb circular RNA [9]. During the late 1990s and early 2000s, several other studies showed that circRNA-producing genes are widespread in eukaryotic cells from flies to mammals including humans [10–18].

Nevertheless, the lack of evidence of their translation into polypeptides left researchers skeptical about the functional significance of such RNAs, and for several decades circRNAs were commonly disregarded as mis-splicing artifacts or by-products of pre-mRNA processing [19]. With the advent of RNA sequencing (RNA-seq) technologies and bioinformatics, the true abundance of circRNAs was revealed. In 2012, an unexpected number of human genes were reported to express “scrambled exons” resulting in circular RNA isoforms [20]. A subsequent analysis of non-polyadenylated libraries prepared from ribosomal RNA-depleted RNAs, revealed >25,000 distinct RNA species containing “non-colinear exons” in human fibroblasts [21] and, at the same time, a new genome-wide *in-silico* approach identified ~2000 human, 700 nematode and 1900 mouse circRNAs that were more stable than the associated linear mRNAs in vivo [18].

**Fig. 1:**
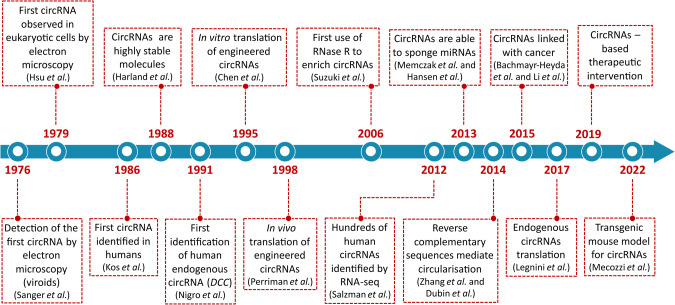
The discovery of circRNAs. Timeline of milestone events leading to the discovery, research and development of circRNAs.

Since their discovery, thousands of circRNAs have been found to be present in multiple organisms and their expression been associated with developmental stages, physiological conditions, and diseases including cancer. This has opened up a new field of study aimed at elucidating their biogenesis and function as an essential part of gene expression programs across eukaryotes (Fig. 1).

## Biogenesis of circRNAs in normal and pathological contexts

### General features of circRNAs

**Fig. 2:**
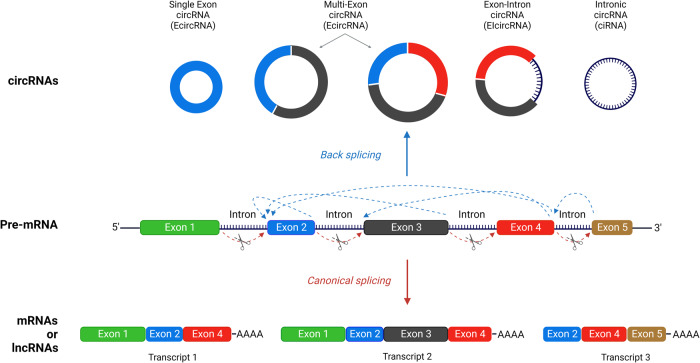
Canonical splicing vs back-splicing. Starting from the same pre-mRNA molecule, linear splicing (red arrows) and head-to-tail back-splicing (blue arrows) lead to a differential outcome in either processed mRNA/lncRNAs molecules or several types of circRNAs, respectively. Adapted from “RNA Processing in Eukaryotes 2”, by BioRender.com (2023). Retrieved from https://app.biorender.com/biorender-templates.

Pre-mRNA splicing, facilitated by the spliceosome, is a mechanism that is tightly coupled with transcription [22]. During this process, introns are removed from precursor mRNAs (pre‐mRNAs) and the exons are covalently joined to create linear mRNA molecules that are translated into proteins. By contrast, back-splicing allows a downstream splice donor (5′ splice site) to join backwards to an upstream splice acceptor (3′ splice site), resulting in a closed continuous head-to-tail molecule known as a circRNA (Fig. 2). The resulting molecules are devoid of terminal 5’ caps and 3′ polyadenylated tails [23–25], are less accessible to exonucleases and are consequently more stable than linear RNAs [26]. By rearranging the order of genomic information, circRNAs also provide a unique opportunity to further diversify gene expression across eukaryotes.

Although circRNAs are preferentially back-spliced from hundreds of human genes [20], including neuron-expressed genes [27], back-splicing is typically much less efficient (<1%) than canonical splicing [28] so circRNAs are often expressed at lower levels than their corresponding linear mRNAs [29]. The observed abundance at levels that exceed mRNAs (frequently over tenfold) in a given cell type [21] is largely due to a context- and time-specific accumulation of circRNAs that occurs for example during aging [30–33] and neurodegenerative [30, 34–36] processes. This is primarily attributed to their exceptional stability. Indeed, whilst mRNAs are continually degraded over time, circRNAs persist, thereby increasing the ratio of circRNA to mRNA as time passes. Packaging into exosomes and subsequent release into the extracellular space [37–40], appears to clear the excessive accumulation of circRNAs avoiding potential toxic effects. In some cases, after being released into the extracellular environment, exosomes can reach recipient cells and deliver circRNAs to trigger functional responses and potentially induce a series of phenotypic changes [41], including the spread of premetastatic niches from cancer-derived exosomes [42, 43]. By contrast, in cancer and highly proliferative cells, circRNAs are maintained at low levels, possibly as a result of dilution effects caused by cell division during the process of cell proliferation [44, 45].

The maturation of circRNAs is a tightly regulated process [46], involving the same spliceosome components engaged in canonical splicing, albeit recruited in a different order [47]. When pre-mRNA processing is inhibited, the intimate connection that exists between canonical splicing and back-splicing [25, 27] results in the splicing machinery facilitating back-splicing and thereby shifting the output of genes toward circRNAs [48, 49]. Additional factors, including epigenetic changes, can impact the biogenesis of circRNA making the expression of mRNA and circRNA at the same locus less predictable. For example, the repression induced by DNA methylation in the main gene body of the parental genes [50, 51] has been found to be one of the causes of altered circRNA expression independent of the linear counterpart [22, 50, 52]. Transcriptional silencing of circRNAs caused by hypermethylation or altered histone modifications at their host gene promoters has also been reported in cancer [53–55].

### Mechanisms of circRNA biogenesis

**Fig. 3:**
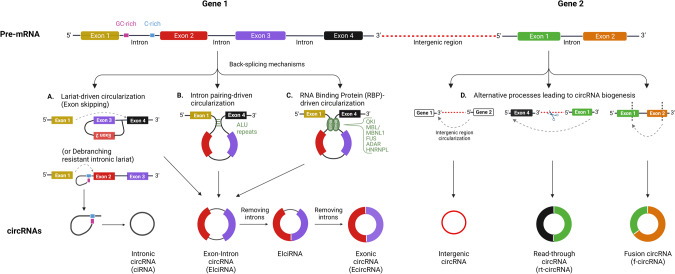
Mechanisms leading to the biogenesis of circRNAs. Three main mechanisms of back-splicing, such as lariat-driven circularization (exon skipping) (**A**), intron pairing-driven circularization (**B**), and RNA binding protein (RBP)‐driven circularization (**C**) lead to the biogenesis of circRNAs. Alternative processes (**D**) can also lead to the biogenesis of other special classes of circRNAs, including intergenic circRNAs or rt-circRNAs and f-circRNAs, which are frequently associated with pathological conditions. Created with BioRender.com.

A variety of circRNAs can be generated from a single protein-coding or non-coding genomic locus [18] (Table 1) and can significantly vary in length since not only are entire exons susceptible to circularization, but so are other sequences, including introns, non-coding antisense, 3′ UTR, 5′ UTR, or transcribed intergenic regions. Additional circRNAs can also be assembled from a combination of multiple exons and retained intronic portions [18, 56]. Generally, at least three, often overlapping [57], mechanisms can lead to back-splicing including lariat-driven circularization (exon skipping) [21] (Fig. 3a), intron pairing-driven circularization [21] (Fig. 3b), and RNA binding protein (RBP)‐driven circularization [27] (Fig. 3c).

**Table 1:** CircRNAs classification.

Category	Abbreviations	Cellular compartment	Mechanism of biogenesis	Ref
Exon-derived circRNAs	ecircRNA	Cytoplasm	1–5 exon circularization	[18, 20, 21]
Exon–intron circRNAs	EIciRNA	Nucleus	Intronic excision failure followed by intronic retention in the nascent circRNAs	[68]
Intronic circRNAs	ciRNA	Nucleus	Intron lariats escaping the usual intron debranching and following degradation	[56, 77, 78]
Intergenic circRNAs	/	/	From intergenic genomic portions and formed by two intronic circRNA fragments flanked by GT-AG (CT-AC) splicing signals acting as the splice donor and acceptor of the circular junction in the process of forming an integrated circRNA	[18, 80]
Read-through circRNAs	rt-circRNA	Cytoplasm	Read-through of polymerase that includes exons from adjacent genes	[49, 81]
Fusion-circRNAs	f-circRNAs	Cytoplasm/ Nucleus	Cancer-associated chromosomal translocation	[83, 84]

In exon skipping a pre-mRNA is spliced into two RNA molecules consisting of an mRNA from which at least one exon is missed, and a lariat containing the skipped exons making circularization possible. The predominant fraction of circRNAs (over 80%) are known to be exon-derived circRNAs (ecircRNAs) [18, 20, 21]; these usually originate from one to five pre-mRNA exons and preferentially localize to the cytoplasm [56], most likely by escaping the nucleus during mitosis [58]. The ATP-dependent RNA helicase DDX39A and the spliceosomal RNA helicase DDX39B are involved in exporting circRNAs from the nucleus to the cytoplasm by sensing the lengths of mature circRNAs by an unclear mechanism [59]. Exonic circularization seems to be strongly favored by longer exon length (generally > 300 nt) [20, 21] flanked by intronic regions of similar length and hosting inverted repeated sequences (e.g., ALU elements) [21, 60, 61]. This appears to occur more frequently through the second mechanism of intron pairing [30] where perfect complementarity between the two introns flanking the exon/exons of the nascent circRNA is required. The successful intron pairing brings the two splice sites into close proximity and the resulting secondary structure facilitates back-splicing [60, 62–64]. Although favorable, the presence of intronic repeat sequences is not always associated with exon circularization [65, 66], and excessive stability of intron base pairing can even sometimes prevent circRNA formation [62].

Several RBPs, including quaking (QKI) [67], muscleblind (MBL/MBNL1) [68], and fused-in-sarcoma (FUS) [69] proteins, participate in circRNA biogenesis by tethering specific motifs within adjacent introns of nascent circRNAs and connecting the splice donor and acceptor sites to form a closed intronic-paired RNA. In the cancer context, QKI has been shown to induce the production of up to one-third of the 300 most abundant circRNAs during epithelial-to-mesenchymal transition (EMT) in immortalized human mammary epithelial cells [67]. CircRNA formation *via* back-splicing is also mediated by heterogeneous nuclear ribonucleoprotein L (HNRNPL) which is required for prostate cancer growth in vitro and is aberrantly expressed in human prostate tumors [70]. In this regard, HNRNPL also facilitates the expression of an oncogenic circRNA from the tumor-suppressor gene *ARHGAP35* generally associated with poor survival in cancer patients [71]. Further mechanisms may combine the intron pairing process involving RBPs with the additional involvement of ADAR (RNA editing) proteins that couple A-to-I editing with the unwinding of the dsRNA helical structures [72], preventing the pairing/looping of intron sequences [73, 74]. By contrast, proteins such as NF90 and NF110 [75], can stabilize the intronic RNA pairs at exonic junctions of nascent circRNAs in response to viral infection and favor their production, which is ultimately reduced when the proteins migrate to the cytoplasm and viral infection terminates [76]. Overall, a combination of both *cis*- and *trans*-acting factors are likely to provide a more complex set of processes that affect circRNA biogenesis [57].

There is added complexity in the formation of circRNAs. When introns are not excised properly and are retained in the newly generated circRNAs, so-called exon–intron circRNAs (EIciRNAs) are generated [68]. Conversely, if intron lariats that are correctly circularized at the branchpoint 2′–5′ linkage and degraded from the 3′ end up to the branchpoint, somehow escape the usual intron debranching and subsequent degradation, stable circular intronic RNAs (ciRNAs) can also be formed [56, 77]. Specific sequences (a consensus motif containing a 7 nt GU-rich element and a 11 nt C-rich element) near the 5′ splice site and branchpoint close to the branchpoint site can prevent debranching by forming a structure that limits access to the debranching enzyme. Both ciRNAs and EIciRNAs are predominantly located in the nucleus and presumably involved in the regulation of expression of neighboring genes in *cis* [56, 68] as they have been found to be associated with RNA polymerase II [56, 78, 79]. Screening of the human transcriptome with a bioinformatic tool for circRNA identification has also revealed the existence of a class of non-exonic circRNAs (intergenic circRNAs). These circRNAs originate from intergenic portions of the genome and contain two intronic circRNA fragments flanked by GT-AG (or reverse complementary dinucleotides CT-AC) splicing signals that act as the splice donor and acceptor of the circular junction while forming an integrated circRNA [80]. Other than a weak but significant enrichment of conserved nucleotides between a few ciRNAs and intergenic circRNAs [18], very little is known about the function of intergenic circRNAs (Fig. 3d).

### CircRNA biogenesis during pathological processes

Distinct classes of circRNA can also be generated in pathological contexts including cancer (Fig. 3d). For instance, the failure of transcription termination and exceeding transcription into the downstream gene followed by back-splicing can give rise to so-called read-through circRNAs (rt-circRNAs) [49, 81] that may incorporate exons from adjacent and similarly oriented genes and appear to be associated with pathological phenotypes [82]. Uncontrolled gene transcription leading to pervasive transcription read-through is typically associated with cancer. Likewise, fusion-circRNAs (f-circRNAs) have been recently reported as originating from cancer-associated chromosomal translocations and are able to confer resistance to apoptosis-inducing drug therapy, promote transformation and cell survival [83, 84]. For instance, the circRNA generated by the *MLL/AF9* fusion gene (f-circM9) in leukemia causes pro-proliferative and pro-oncogenic effects [84]. Similarly, the back-splicing of the non-small cell lung cancer (NSCLC)-associated *EML4-ALK* fusion variant 3b generates a tumor-promoting circRNA named f-circEA [83] and an additional variant (f-circEA-2a) which enhances cell migration and invasion [85].

### Further complexity in circRNA biogenesis

Some circRNAs may also contain modified nucleotides, such as N6-methyl-adenosine patterns (m6A), that can further diversify their biogenesis [86–88], as well as their fate, including their degradation and cellular localization [89]. These m6A-modified circRNAs often originate from unmethylated exons of linear mRNAs and they are likely to be methylated during or after circRNA formation [40, 88]. According to recent studies, the efficient depletion of specific enzymes involved in such modifications (e.g., methylation writers, readers, etc.) affects a subset of circRNAs (~20%) without significantly altering their linear isoforms [90]. However, it is unclear whether or not additional factors play a role in the mechanism through which m6A deposition may affect the choice of back-splicing versus canonical splicing.

Alternative explanations of back-splicing should also be contemplated. A proportion of discovered circRNAs might not be functional per se but might offer through their back-splicing a break between transcription of the main gene and translation, allowing post-transcriptional regulatory processes to take place. Offering a more radical perspective, recent studies have also proposed that circRNAs form through pre-mRNA splicing errors and are not able to confer any specific benefit [91].

## Biological functions and mechanisms of action of non-coding and translated circRNAs

The number of unique circRNAs produced in human cells (~100,000) [81, 92] largely exceeds the number of protein-coding genes (~20,000) [93–95]. Despite their wide prevalence, the majority of circRNAs have not been functionally characterized and the biological role of many remains unclear [15, 16, 91]. A growing body of evidence suggests that those carrying out biological functions are likely to require a specific subcellular localization and that their accumulation in specific disease contexts [18], may indicate a link with the occurrence and development of specific diseases including cancer. Despite the fact that the majority of circRNAs are spliced out from protein-coding pre-mRNAs, they are mainly classified as a special class of non-coding RNAs (ncRNAs) since they are devoid of essential elements for translation such as an open reading frame (ORF) and a 5′ cap and the poly(A) tail, and are characterized by an average length frequently longer than 200, that occasionally (e.g., in the case of ecircRNAs and ciRNAs) can shorten to 100-200 nt [9].

### CircRNAs as regulators of transcription

**Fig. 4:**
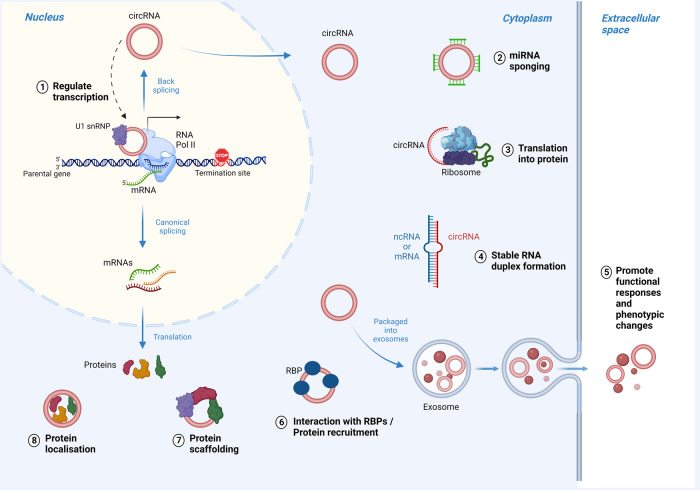
Biological functions of circRNAs. CircRNAs can work as multifunctional devices serving as transcription regulators of their parental genes (1), as microRNA sponges affecting genes post-transcriptionally (2), or as translated short peptides/proteins (3). Additionally, circRNAs can affect the stability of other RNA molecules (mRNAs or lncRNAs) (4), accumulate inside of exosomes and mediate cellular response (5), engage with RBPs and acting as either decoy (6), or scaffold molecules (7), as well as directing RBP cellular localization (8). Adapted from “DNA vs mRNA Transfection”, by BioRender.com (2022). Retrieved from https://app.biorender.com/biorender-templates.

Beyond their regulatory effects on alternative pre-mRNA splicing [27], circRNAs can function in more than a single mechanism (Fig. 4, Table 2), including as regulators of transcription of the same genes from which they are transcribed (parental genes) either alone or in association with RBPs. In this regard, they have been found to either upregulate the expression of specific transcription factors and activate their parental gene’s transcription, or to favor premature transcription termination through the formation of RNA-DNA hybrid (R-loops) with the subsequent upregulation of a truncated, non-functional isoform [96]. CircRNAs can also induce promoter CpG demethylation thereby changing the epigenetic state, and switch on the activity of their parental genes [97, 98]. Through this positive feedback mechanism of inducing DNA hypomethylation in CpG islands of the promoter by recruiting the methylcytosine dioxygenase TET1, circFECR1 activates its parental *FL1* (friend leukemia virus integration 1) (onco) gene and favors breast cancer cell metastasis [98]. An interaction with the normal activity of RNA polymerase II and with other components of the transcription machinery proteins has also been reported [56, 68]. CircRNAs that are mainly located in the nucleus, such as EIciRNAs, can bind the small nuclear U1 ribonucleoprotein (U1 snRNP) through RNA-RNA base pairing and then interact with RNA polymerase II at the parental gene promoter thereby enhancing their expression [68]. Similarly, ciRNAs accumulate at their sites of transcription and can increase the transcription rate of parental genes by tethering the elongation Pol lI complex and ultimately regulating its elongation activity [56].

**Table 2:** Mechanisms of action of circRNAs in human cancers.

CircRNA	Cancer type	Role	Mechanism of Action	Refs
circHIPK3	Lung cancer	Activates PI3K/AKT signaling pathway	Sponging miR-188-3p	[224]
circTP63	Lung cancer	Facilitates cell-cycle progression	Competitive binding to miR-873-3p	[225]
circFGFR1	Lung cancer	Upregulates CXCR4, anti-PD-1 resistance	Sponging miR-381-3p	[203]
circFOXM1	Lung cancer	Suppresses FAM83D	Sponging miR-614	[226]
circFARSA	Lung cancer	Upregulates B7-H3	Sponging miR-15a-5p	[227]
circXPO1	Lung cancer	Promotes metastasis	Binding to IGF2BP1 and stabilizing *CTNNB1*	[110]
circNDUFB2	Lung cancer	Acts as a tumor suppressor and favors antitumor immunity	Forming complexes with TRIM25 and IGF2BPs for IGF2BPs ubiquitination	[118]
circNSUN2	Colorectal cancer	Promotes malignant tumor progression	Binding to IGF2BP1 and stabilizing *HMGA2* mRNA	[89]
circPPP1R12A	Colon cancer	Promotes cell proliferation and metastasis	Encoding circPPP1R12A-73aa and regulates the Hippo-YAP signaling	[128]
circYAP	Breast cancer	Inhibits YAP translation	Binding p53 and MDM2	[228]
circDNMT1	Breast cancer	Induces autophagy	Nuclear translocating AUF1, p53	[210]
circFOXO3	Breast cancer	Promotes PUMA expression and apoptosis	Facilitating p53 ubiquitination	[229]
circSKA3	Breast cancer	Promotes cell invasion	Interacting with Tks5 and integrin β1	[230]
circWWC3	Breast cancer	Promotes EMT, upregulates EGFR, MAPK1	Sponging miR-26b-3p, miR-660-3p	[231]
circTADA2A	Breast cancer	Suppresses migration and invasion	Sponging miR-203a-3p	[232]
circFECR1	Breast cancer	Promotes metastasis	Activating *FL1* parental gene	[98]
circNRIP1	Gastric cancer	Activates AKT1/mTOR pathway	Sponging miR-149-5p	[233]
circSERPINE2	Gastric cancer	Activates BCL2 signaling pathway	Sponging miR-375	[234]
circDONSON	Gastric cancer	Promotes cell migration *via* SOX4	Binding BURF complex	[235]
circDLST	Gastric cancer	Activates NRAS/MEK/ERK1/2 pathway	Sponging miR-502-5p	[236]
circLMTK2	Gastric cancer	Acts as a tumor suppressor	Sponging miR-150-5p	[237]
circMAPK1	Gastric cancer	Acts as a tumor suppressor	Encoding MAPK1-109aa which suppresses MAPK signaling	[238]
circDLDG1	Gastric cancer	Promotes EMT, metastasis and anti-PD-1 resistance	Regulating CXCL12 by sponging miR-141-3p	[204]
circARHGAP35	Hepatocellular carcinoma	Promotes tumor progression, invasion and metastasis	Encoding circARHGAP35 protein which interacts with the transcription factor TFII-I	[71]
circNT5E	Glioma/Gliobastoma	Upregulates NT5E, SOX4 and PIK3CA	Sponging miR-422a	[239]
circNFIX	Glioma/Gliobastoma	Activates Notch signaling	Sponging miR-34a-5p	[240]
circTTBK2	Glioma/Gliobastoma	Upregulates EZH2	Binding miR-217/HNF1β/Derlin-1	[241]
circCFH	Glioma/Gliobastoma	Regulates the AKT signaling	Sponging miR-149	[242]
circSMARCA5	Glioma/Gliobastoma	Acts as a competitive endogenous RNA	Binding SRSF1/SRSF3	[243]
circE-cad	Glioma/Gliobastoma	Maintains cancer stemness	Encoding C-E-Cad- 254aa that interacts with EGFR and activates STAT3 signaling	[129]
circFBXW7	Glioma/Gliobastoma	Inhibits the effects on cell cycle	Encoding FBXW7-185aa which causes reduction of c-Myc half life	[127]
circSHPRH	Glioma/Gliobastoma	Acts as a tumor suppressor	Encoding SHPRH-146aa	[126]
circPAN3	Acute myeloid leukemia	Mediates drug resistance via AMPK/mTOR	*Via* miR-153-5p/miR-183-5p-XIAP axis	[244]
circDLEU2	Acute myeloid leukemia	Promotes PRKACB expression	Suppressing of miR-496	[245]
circPVT1	Acute myeloid leukemia	Regulates cells proliferation, migration and apoptosis	Stabilizing c-MYC and CXCR4 expression	[246]
circHIPK2	Acute myeloid leukemia	ATRA-induces differentiation	*Via* miR-124-3p/CEBPA	[247]
circRNF220	Acute myeloid leukemia	Increases cell migration, invasion	Sponging miR-30a	[248]
circHIPK3	Chronic myelocytic leukemia	Increases cell proliferation and invasion	Sponging miR-124, miR-506	[249]
circBA9.3	Chronic myelocytic leukemia	Promotes cancer cell survival	Upregulating c-ABL or BCR-ABL1	[250]
circITCH	Multiple myeloma	Acts as a tumor suppressor	Regulating miR-615-3p/PRKCD axis	[249]
circIARS	Pancreatic cancer	Promotes metastasis *via* exosomes	Downregulating miR-122	[251]
circPDE8A	Pancreatic cancer	Promotes tumor invasion	*Via* miR-338/MACC1/MET axis	[252]
circHIPK3	Esophageal squamous cell carcinoma	Regulates cell growth	Sponging miR-124	[106]
circITCH	Esophageal squamous cell carcinoma	Acts as a tumor suppressor	Sponging miR-7, miR-17, miR-214, etc	[107]
circPABPN1	Cervical carcinoma	Regulates translation of parental gene *PABPN1*	Binding RBP HUR	[116]

### CircRNAs as miRNA sponges

Initial examples of functional circRNAs were shown to work as efficient microRNA sponges, post-transcriptionally regulating the activity of their downstream target genes [99], as well as acting as miRNA-reservoirs or miRNA-cargos. This has been widely confirmed by their significant localization inside exosomes [40] and a well reported association with Argonaute proteins [99]. Since the discovery of the antisense transcript cerebellar degeneration-related protein (CDR1as), also known as the circRNA sponge for miR-7 (CiRS-7), and its role as a competing endogenous RNA (ceRNAs) [99] with > 60 miRNA binding sites, more studies have corroborated the ability of circRNAs to act as ceRNA or miRNA sponges [100, 101]. Among several circRNAs associated with altered target gene expression in cancer [102–105], circHIPK3 regulates cell growth by sponging multiple miRNAs, such as the tumor-suppressor miR-124 [106], whereas circITCH acts as cancer inhibitor by sponging several miRNAs, including miR-7, miR-17, and miR-214, suppressing the Wnt/β-catenin pathway in esophageal squamous cell carcinoma (ESCC) [107].

### CircRNAs as modulators of RNA stability

In addition, circRNAs can influence the stability of other RNA molecules, including both lncRNAs and mRNAs [108]. An example is the case of CiRS-7/CDR1as which can stabilize its cognate mRNA by forming an RNA duplex [17]. The stabilization of RNA molecules can also occur in cooperation with proteins; for example, circZNF609 favors the recruitment of the protein ELAV1 (also known as HuR), and its interaction increases the stability and/or translation of a pool of mRNAs, including *CKAP5* mRNA expressing a protein that regulates microtubule function and sustains cell-cycle progression in cancer cells [109]. Likewise, circXPO1 promotes lung adenocarcinoma (LUAD) progression by recruiting IGF2BP1 to enhance the stability of *CTNNB1* mRNA [110]. A similar mechanism was described a few years earlier in colorectal cancer for circNSUN2 which is able to stabilize *HMGA2* mRNA by enhancing its interaction with IGF2BP2 to promote malignant progression [89].

### CircRNAs as RBP partners

As well as being required for their back-splicing and gene transcription regulation, circRNAs are also able to engage with RBPs to direct their cellular localization [111]. Given their preferential location inside the cytoplasm, circRNAs can indeed sequester cytoplasmic proteins and prevent their nuclear entry [112], serve as RBP decoys to regulate their function [113] or act as scaffold molecules for complex assembly [55, 114, 115]. For instance, in human cervical carcinoma HeLa cells, circPABPN1, binds the RBP HuR and affects the translation rate of the parental gene *PABPN1* by preventing HuR binding to the cognate mRNA [116]. In this regard, a remarkable tumor-suppressor example in breast cancer is represented by the circFOXO3 that binds both p53 and the E3 ubiquitin ligase MDM2, which normally mediates the degradation of the transcription factor Foxo3. This association promotes the degradation of P53 while preventing the degradation of the pro-apoptotic Foxo3 derived from its parental gene [117]. In NSCLC, the inhibitory effects on tumor growth and metastasis are instead caused by the scaffolding action of circNDUFB2 that forms a ternary complex with both TRIM25 and IGF2BPs to facilitate the ubiquitination and degradation of IGF2BPs and ultimately activate an antitumor immune response [118]. Conversely, the successful interaction of the oncogenic circ-Amotl1 with the proto-oncogene *c-MYC* ensures the retention in the nucleus of c-Myc protein and consequently promotes tumorigenesis [119].

### CircRNAs as translated peptides

Although the majority of circRNAs are expected to be non-coding, both in vivo and in vitro experiments have demonstrated their association with ribosomes and translation into proteins [14, 120, 121]. Bioinformatic predictions estimate that only a small proportion of circRNAs host both ORFs and internal ribosome entry site (IRES) elements, or incorporate the m6A RNA modification in their 5′ UTR thereby becoming competent for translation *via* a cap-independent mechanism. As a result, shortened versions of canonical proteins, acting as modulators of dominant negative protein variants, decoys, or alternative protein complexes are generated [120]. However, according to a recent study, IRES-like short elements that are significantly enriched in endogenous circRNAs, are sufficient to drive extensive circRNA translation [122], suggesting that circRNA translation might be a far more widespread phenomenon than initially estimated [123–125]. Besides the better studied translated circZNF609, which harbors IRES elements and undergoes cap-independent translation [120], cancer-associated examples of translated circRNAs include the circular form of the *SHPRH* gene (circSHPRH) which encodes the novel identified protein termed SHPRH-146aa [126]. Together with circSHPRH, SHPRH-146aa is normally highly expressed in normal human brains but downregulated in glioblastoma, suggesting a potential role as a tumor suppressor [126]. Inhibitory effects on glioma proliferation and cell-cycle acceleration are instead mediated by a 185 amino acid protein encoded from circFBXW7 (FBXW7-185aa) which reduces the half life of c-Myc by antagonizing USP28-induced c-Myc stabilization, suggesting new prognostic implications for glioma patients [127]. By contrast, translation of the circPPP1R12A, (generally up-regulated in colon cancer) into a 73 amino acid small polypeptide (circPPP1R12A-73aa) promotes rapid cancer cell proliferation and metastasis *via* the Hippo-YAP pathway [128]. A similar oncogenic effect is caused by the E-cadherin variant encoded by the circE-Cad at the *CDH1* gene (C-E-Cad- 254aa), involved in the maintenance of the cancer stemness in glioblastoma by interacting with EGFR and activating downstream STAT3 signaling [129]. In hepatocellular carcinoma (HCC), tumor progression, invasion and metastasis, can instead be caused by an exceptionally long (1289aa) oncogenic protein, encoded by circARHGAP35 through alternative m6 A-dependent translation [130] that interacts with the transcription factor TFII-I in the nucleus [71]. Oncogenic virus-derived circRNAs can also be translated into proteins. For example, the highly expressed human papillomavirus-derived circE7, displays oncogenic activity in cervical and head and neck cancers, and has been found to be translated into an E7 oncoprotein [131]. Experimentally validated and functionally characterized peptides encoded by ncRNAs (ncPEPs), including circRNAs, have been recently annotated in the new database FuncPEP [132].

## Detection and characterization of circRNAs: challenges and strategies

Clinical applications of circRNAs rely on accurate RNA profiling (Box 1); this includes annotating new RNA species and quantifying their abundance [133]. However, detecting and studying circRNAs poses challenges at several levels due to their circular conformation and sequence overlap with their linear mRNA counterparts.

Box 1 Chapter: Step-by-step methods for investigating circRNAs: from discovery to function It’s worth noting that different methods have their own advantages and limitations. Combining multiple approaches can enhance the reliability and accuracy of circRNA detection and validation.

1. Initial identification and enrichment
  **RNAse R treatment**: An exoribonuclease that specifically degrades the bulk of linear transcripts and enriches circRNAs in a K^+^ containing buffer [21, 137].
  **A-tailing + RNAse R treatment:** Purified RNA samples are treated with poly(A) polymerase followed by RNase R treatment in a Li^+^ containing buffer, to remove linear RNAs more efficiently [253].
  **Microarrays**: Reverse transcribed RNAs (cDNAs) hybridized with probes immobilized on to microarray chips that target circRNA back-splice junctions [140].
  **Northern Blotting**: Separation of linear and circular RNA molecules based on electrophoretic mobility, immobilized on a nitrocellulose membrane and targeted by complementary radiolabeled or non-radiolabeled oligonucleotide probes [139].
  **RNA-seq libraries and deep sequencing:** Transcriptome-wide profiling of circRNAs.
2. Validation of sequence, presence, and relative abundance
  **RT-qPCR**: cDNAs are amplified and quantified with “divergently oriented” primers designed across the junction sequences.
  **Sanger sequencing**: Conventional sequencing to confirm the circular nature of an RNA molecule once amplified.
  **RT-RCA:** Reverse transcription by rolling cycle amplification using primers binding to the circRNA junction site [153].
  **RNA-FISH:** Fixed cells or tissues are hybridized with short fluorescently labeled DNA probes targeting back-splice junctions of a circRNA [145, 146].
  **LAMP:** Double exponential amplification induced by employing stem-loop primers (SLPs) that recognize the junction sequence of circRNAs and form a double stem-loop DNA structure [154].
  **Nanostring technology (nanoString nCounter):** Capture and reporter color-coded probes that jointly recognize the back-splice junction of a circRNA [162].
3. Modulating circRNA levels
  **Silencing systems:** Targeting the junction region of the circRNAs (siRNAs, shRNAs, antisense oligonucleotides (AONs), CRISPR–Cas13 [155, 157], *etc*).
  **Overexpressing systems:** To increase circRNA expression (plasmids, viral vectors, transgenic models, *etc*).
4. Identification of partner molecules (RBP or miRNAs)
  **RIP**: Antibody pulldown of a circRNA directly bound to a specific protein of interest.
  **Biotinylated AONs:** Oligonucleotides designed against the circRNA junction to pull down the circRNA.
5. Assessing circRNA stoichiometry and absolute quantification
  **RT-qPCR:** Using a spike external RNA at a known concentration followed by RT-qPCR to generate a standard curve correlating Ct values with copy number.
  **Droplet digital PCR (ddPCR):** Droplets with single cDNA molecules are amplified individually allowing absolute quantification of circRNA copies per input RNA [151].
6. Validating the functional consequences of circRNA interactions
  **Rescue experiments:** Comparing the biological effects observed from mutated or truncated circRNAs with those of endogenous circRNAs and overexpressed circRNAs.

### Methods for the detection of circRNAs

RNA-based high-throughput sequencing technologies (e.g., RNA-seq), have allowed genome-wide annotation and quantification [133] of a number of diverse coding and non-coding RNAs [134, 135] based on the selective isolation of ribosomal RNA (rRNA)-free polyadenylated RNA species using oligo-dT primed reverse transcription. Improved methodologies that employ the preparation of rRNA-depleted libraries and random priming for cDNA synthesis (ribo-depleted total RNA-seq) [135], have enhanced the annotation of RNAs to include large non-polyadenylated transcripts encompassing a compendium of circRNAs [136].

Traditional methods, such as RT-qPCR using sets of “divergently oriented” primers designed to cover the circRNA back-spliced junction, and Northern blotting, which can separate RNA molecules of different size and abundance based on their speed in electrophoresis [25, 30, 34], can help to discern individual circRNAs from their linear counterparts. RNase R treatment-based strategies that preferentially degrade linear over circular RNAs, have been used to validate the presence of and enrichment of circRNAs in a total RNA pool [21, 137]. However, the real efficacy of such techniques remains controversial [45, 138] as demonstrated by one of the best characterized circRNAs, CiRS-7/CDR1as, which is exceptionally sensitive to RNase R treatment [21]. These conventional methods provide useful but limited information and, as in the case of Northern blotting, they are limited by low sensitivity, low throughput, and elaborate steps [139].

The use of microarrays with probes spanning back-splice junctions has also been employed as a suitable screening tool; however, this approach can sometimes lead to the erroneous detection of linear species and can generate data that is not easily consistent between studies [140]. Nonetheless, the identification and quantification of circRNAs requires especially designed bioinformatics pipelines. Over the years, several computational algorithms have been developed to detect non-linear splice events by sequence alignment of reads covering the back-splice site to the reference genome, including algorithms that can reconstruct full-length circular RNAs [141]. Differences in their sensitivity and precision can unfortunately lead to an underestimation and incomplete annotation of circRNAs [136, 142]. A similar challenge applies for both de novo and exon–intron sequence-based prediction tools that can often generate dramatic differences in output and therefore require further validation with other approaches [143]. A comprehensive evaluation of ~100 circRNA bioinformatics tools, including web services, databases, stand-alone programs and pipeline tools based on their performance and limitations, has been recently published [144].

Intracellular visualization, localization, and quantification of RNA molecules are critical for studying their biology and function. Several studies have employed RNA fluorescence in situ hybridization (RNA-FISH) [145] for the quantification and localization of circRNAs, including CiRS-7/CDR1as [146]. RNA-FISH has also been used to confirm the colocalization of circRNA and target miRNAs, such as CiRS-7/CDR1as and miR-135a in bladder cancer [147], circRHOBTB3 and miR-654-3p in gastric cancer cells [148], circFAM114A2 and miR-762 in urothelial bladder carcinoma [149]. However, probe designs that can specifically and uniquely target the back-splice junctions of circRNAs can often be challenging, making the overall technique time-consuming and costly for an efficient signal detection.

Advances in the field of circRNA research have paved the way for a number of novel assays with increased sensitivity and specificity that appear to detect more accurately low abundance circRNAs and hold great promise for their efficient annotation in the future [150]. Among several techniques, the recent development of reverse transcription-droplet digital polymerase chain reaction (RT-ddPCR) demonstrates the ability to provide absolute copy numbers and to detect low abundance circRNAs based on partitioning nucleic acids into nanoliter-sized droplets containing the target sequence for PCR amplification [151]. This approach has been successfully employed to profile circRNA levels in plasma. The method has led to the positive correlation between plasma levels of hsa_circ_0001017 and hsa_circ_0061276 with the overall survival of gastric cancer patients [152]. Particularly suitable for profiling circRNAs is the rolling cycle amplification (RCA) method in which a primer can bind to the junction site on the target circRNA and allow the reverse transcriptase to begin rolling cycle amplification. This produces a single-stranded cDNA of a long-chain of hundreds of repetitive fragments that amplify the signal [153]. A similar strategy based on pairs of stem-loop primers (SLPs) that recognize the junction sequence of circRNAs and form a double stem-loop DNA structure, can induce double exponential amplification (LAMP) and specifically detect circRNAs from linear RNAs [154].

### Methods for the functional characterization of circRNAs

Beyond efficient detection, studies aimed at assessing circRNA function require particular attention. Albeit at different levels, circRNAs and linear RNAs transcribed at the same genomic locus are normally co-expressed. This makes gain- and loss-of-function studies particularly challenging since they are based on targeting the original loci and are therefore likely to affect the cognate linear RNAs too. In terms of selective degradation of circRNAs, of particular note is the newly developed RNA-guided, RNA-targeting Cas13 system (CRISPR–Cas13) [155, 156], which has been shown to be far more efficient and specific in circRNA knockdown, compared to standard RNAi approaches [157, 158]. By designing guide RNAs that target sequences spanning the back-splice junction, CRISPR–Cas13 can knock down circRNAs, without any impact on related linear mRNAs. This has enabled the efficient and specific knockdown of the oncogenic circFAM120A, which normally promotes cell proliferation by efficiently favoring the translation of its cognate linear mRNA (*FAM120A*) by competitively binding to IGF2BP2 (a translation inhibitor) [159]. Strategies based on expression vectors that drive almost exclusively circular and not linear exons [48] by employing flanking introns with base-pairing repeats, have also proven to be, with appropriate controls, hugely advantageous in studying new circRNA functions [62]. A successful genetically engineered mouse model harboring circRNA expression constructs, has recently been employed to study circRNAs in vivo in the context of melanoma [160].

Cutting-edge methodologies, such as the new Oxford Nanopore technology, are particularly suitable for long-read sequencing and may further help in providing information about the entire sequence of circRNAs [161]. Furthermore, the use of NanoString platform offers more accurate detection and quantification of individual circRNAs by using capture and reporter color-coded probes that jointly recognize the back-splice junction without the need for amplification or reverse transcription [162, 163]. For a detailed description of methods used to study and characterize circRNA functions and mechanisms of action, we recommend three excellent recent reviews [164–166].

## CircRNAs as powerful biomarkers

CircRNAs have been linked to a variety of physiological conditions and cell biology features including stemness and pluripotency [44, 167–172] and are therefore potentially implicated in inducing and sustaining cancer development. Moreover, circRNAs have been correlated with some clinical characteristics such as the histological grade, size, metastasis stage and aggressiveness of cancer [163, 173].

**Fig. 5:**
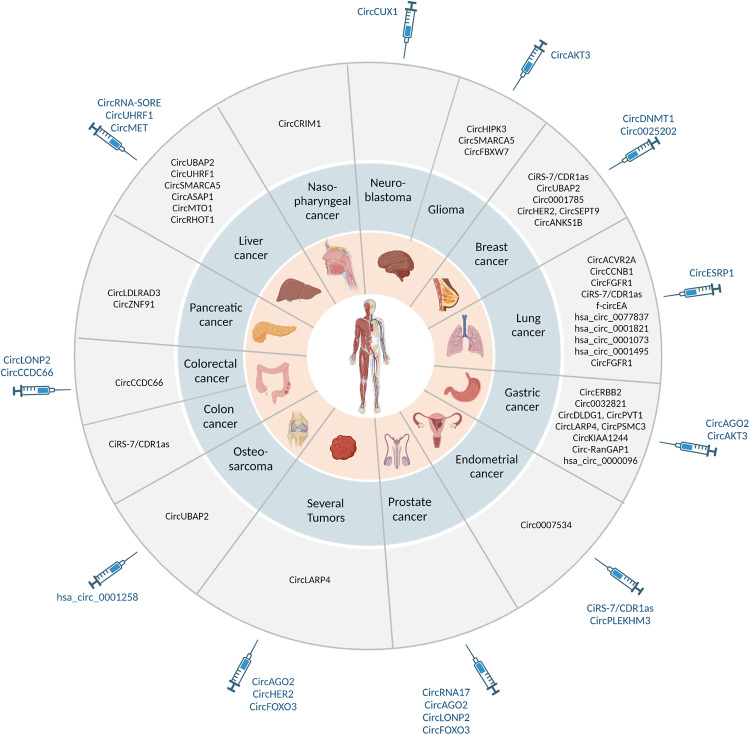
CircRNAs as biomarkers and therapeutic targets in human cancer. An up-to-date summary of circRNAs that show promise as clinical biomarkers (outmost circle) and/or as therapeutic targets (syringe icons pointing towards the chart), associated with different types of cancer. Created with BioRender.com.

Growing evidence suggests that circRNAs can be used as potential biomarkers for early-cancer detection, clinical diagnosis, prognosis and even used in monitoring response to therapy [174–177] (Fig. 5). Their unique expression patterns, molecular stability, specificity and broad distribution across human body compartments, make circRNAs accessible for relatively easy detection and quantification by liquid biopsy in body fluids, including blood, sputum and urine [81, 178]; the latter are more preferable and effective than tissue biopsy due to the minimal invasiveness and feasibility of repetitive sampling.

### CircRNAs as prognostic biomarkers

Some circRNAs have shown a strong prognostic potential including CiRS-7/CDR1as which has been found highly abundant in intratumoral stromal cells, extensively used as prognostic factor in carcinomas of colon [179], lung [180] and breast [181]. A strong potential for predicting gastric cancer prognosis has also been proposed for circERBB2, whose plasma levels in pre-operative gastric cancer patients significantly correlate with the occurrence of lymph node metastasis [177]. Whereas the detection of tumor-suppressor circRNAs such as circLARP4 has been associated with good prognosis in several different cancers [182–185], the detection of oncogenic circUBAP2 has been linked to unfavorable prognosis in HCC, breast cancer and osteosarcoma through the sponging of different miRNAs [159, 186, 187].

CircRNA panels and/or signatures appear to have a more robust prognostic value than single circRNAs. RNA-seq analysis of frozen tissues collected from post-operation has allowed to profile differential circRNA expression between patients with and without recurrence in four circRNA-based signatures (named circScores), thereby grouping colon cancer patients based on high- or low-risk of recurrence [188]. According to the standard of care for younger patients developed by the Nordic Lymphoma Group, the introduction of cytarabine-containing chemoimmunotherapy followed by autologous stem cells transplant increased mantle-cell lymphoma (MCL) patient’s survival. However, treated MCL patients are likely to experience continuous long-term relapse. Analysis on a cohort of samples from two clinical trials conducted by the Nordic Lymphoma Group, has allowed to profile circRNA expression patterns and help with the identification of high-risk disease patients treated with cytarabine-containing chemoimmunotherapy and autologous stem cells transplant [163].

### CircRNAs as diagnostic biomarkers

CircRNA tissue and cell-type specificity allow better correlation with specific pathologies, including the ability to distinguish between cancer subtypes [81, 189–192] and clinical stages. This is particularly beneficial in early-cancer diagnosis and increases the chances of patient survival. In this regard, a group of researchers have developed a unique circRNA test that detects five circRNAs in urine-derived extracellular vesicles and has the potential to distinguish high-grade prostate cancer from benign prostatic hyperplasia [193]. CircRNA expression profiles could also help with the classification of breast cancer subtypes into HER2-positive, estrogen receptor-positive and triple negative breast cancers [191]. Similarly, the detection of low and high level of expression of circACVR2A and circCCNB1 respectively, has enabled the distinction between adenocarcinoma and squamous cell carcinoma in NSCLC [192]. Plasma-based circRNAs assays with high detection accuracy have also allowed the distinction of HCC patients with hepatitis B virus infection from healthy individuals and patients with chronic hepatitis B and liver cirrhosis [194].

Exosome-derived circRNAs are also useful diagnostic biomarkers depending on relative expression, stability, and exosome coupled targeted delivery pathways [40]. For instance, exosomal circRNAs enriched in serum have shown high potential for the early diagnosis of colorectal cancer [40]. An early diagnosis of the most frequent and deadly human brain cancer, glioblastoma multiforme, could instead be possible by monitoring downregulated levels of exosome-derived circHIPK3 and circSMARCA5 [195].

### CircRNAs as both prognostic and diagnostic biomarkers

Some circRNAs could offer a double utility as both prognostic and diagnostic biomarkers. For example, circ0001785 not only shows higher diagnostic efficiency for breast cancer detection than the two most widely investigated biomarkers serum carcinoembryonic antigen (CEA) and cancer antigen 15-3 (CA15-3) [196], but also possesses strong prognostic potential in predicting the histological grade, TNM (Tumor, Node, Metastasis) stage and distant metastasis in breast cancer progression [196]. Similarly, circLDLRAD3 has been proposed as a promising biomarker in both the diagnosis and prognosis of pancreatic cancer since it was found to be elevated in the plasma of patients and strongly correlate with venous and lymphatic invasion [197].

### CircRNAs as predictive biomarkers for cancer therapy

The efficacy of cancer therapy is often limited by intrinsic and acquired resistance. CircRNA expression has been proven to vary in response of tumor cells to chemotherapy, radiotherapy and immunotherapy through several mechanisms, thereby serving as a valuable indicator for clinicians to modify cancer patient treatment. For instance, circCRIM1 competitively binds to miR-422a and prevents the inhibitory effects of miR-422a on its target gene *FOXQ1*, which ultimately leads to metastasis in nasopharyngeal carcinoma, EMT and docetaxel chemoresistance [198]. In endometrial cancer, resistance to paclitaxel, is mediated by a key oncogenic circRNA (circ0007534) which sponges miR-625 and subsequently increases the expression of the miR-625 target gene *ZEB2*, a master regulator of EMT [199]. Interestingly, prostate cancer therapy with androgen receptors inhibitors such as enzalutamide, dramatically changes the expression of a pool of circRNAs in enzalutamide-resistant cells, opening new scenarios for understanding resistance mechanisms and offering novel opportunities for treating prostate cancer patients [200].

Given their circulating nature, exosomes can also transmit drug resistance between heterogeneous populations of tumor cells. CircRNAs with altered expression in drug-resistant cells can be transferred to drug-sensitive ones. For example, the exosomal circZNF91 functions as an exosomal cargo mediating the signal transmission between hypoxic and normoxic tumor cells in pancreatic cancer and promotes chemoresistance [201]. In a similar vein, the exosome-associated circ0032821 promotes oxaliplatin resistance in gastric cancer (GC) cells by regulating SOX9 *via* miR-515-5p [202].

Binding of the cell receptor PD-1 to its programmed death-ligand 1 (PD-L1) on tumor cells, activates downstream signaling pathways and inhibits T cell activation. Novel antibody inhibitors, such as anti-PD-1 and anti-PD-L1, are designed to restore the host antitumor immune response effectively, with an overall manageable toxicity compared to chemotherapy and radiotherapy. However, the development of resistance to immune checkpoint inhibitors (ICIs) is one of the major limitations in using novel anti-PD-1 and anti-PD-L1 therapies for treating cancer. CircRNAs play an important role in acquiring resistance features to these therapies by modulating the expression of key cancer pathways and associated immune populations from the tumor microenvironment. For example, in NSCLC circFGFR1 induces resistance to PD-1 antibodies by interacting with miR-381-3p and upregulating its target CXCR4, responsible for NSCLC progression and resistance [203]. In distant metastatic lesions and gastric cancer tissues, overexpressed circDLDG1 sponges miR-141-3p and increases the expression of CXCL12 which in turn promotes EMT, proliferation, metastasis and resistance to anti-PD-1 inhibitors [204]. Another anti-PD-1 resistance-associated circRNA, is circUHRF1 which is generally overexpressed in HCC tissue, cell lines, and linked with poor prognosis. CircUHRF1 is secreted *via* exosomes by HCC cells into the surrounding microenvironment and by sequestering and preventing miR-449c-5p binding to the checkpoint target gene “T cell immunoglobulin and mucin domain 3” (*TIM-3*), triggers natural killer (NK) cell dysfunction, immune evasion and resistance to anti-PD-1 immunotherapy [205].

## Therapeutic application of circRNAs

The dysregulation of circRNAs can drive tumorigenesis and metastasis in various cancers, making them promising targets for cancer treatment (Fig. 5). Several therapeutic approaches aimed at modifying circRNA expression are currently under investigation. Preclinical studies in animal models are mostly focused on gain- or loss-of-function strategies through circRNA overexpression or knockdown.

### Therapeutic applications based on circRNA knockdown

RNA interference (RNAi) accomplished by cytoplasmic delivery of small interfering RNAs (siRNAs) or short hairpin RNAs (shRNAs) [206] has been used to silence single or multiple circRNAs in vivo. For example, treatment with shRNAs targeting circCUX1, a circRNA generally overexpressed in neuroblastoma (NB) that is able to promote cell proliferation and invasion *via* sponging miR-16-5p, has been shown to reduce tumor growth in mice efficiently [207]. Repression of tumorigenesis in mice has also been shown upon the silencing of circAGO2 [208], a circRNA that is up-regulated in several cancers and associated with poor prognosis. CircAGO2 interacts with HuR protein to facilitate HuR activation and enrichment on the 3’ UTR of target mRNAs, which reduces the accessibility for AGO2 binding and AGO2/miRNA-mediated silencing to mRNAs associated with cancer progression [208].

Single-stranded DNA antisense oligonucleotides (AONs) have also been employed to inhibit selectively or degrade oncogenic circRNAs. For example, AONs targeting circLONP2, which enhances metastasis and invasiveness of colorectal cancer cells by favoring the maturation and the exosomal dissemination of miR-7, dramatically reduce the extent of metastasis to foreign organs in vivo [209].

The delivery of RNAi molecules can be better accomplished with the use of nanoparticles. Gold nanoparticles (AuNPs), commonly associated with a linker such as PEG or polyethylenimine, can be conjugated with siRNAs, shRNAs or AONs and ensure a more efficient drug delivery in animal studies thanks to their high stability, and easily modifiable surface. Delivery of AuNPs with siRNAs targeting circDNMT1 has been shown to suppress breast tumor growth successfully as well as enhancing the survival in mice [210]. A similar approach with AuNP conjugated with AONs aimed to block the binding sites on circCcnb1 for both Ccnb1 and Cdk1, has also shown to inhibit tumor growth and promote mouse survival [211].

Translated circRNAs with important oncogenic roles in cancer can also be targeted by specific drug inhibitors. This is the case of HER2-103aa which plays a role in tumorigenesis and shares sequence similarity with HER2 CR1 domain (the domain that is targeted by pertuzumab). Treatment with pertuzumab has proven to reduce significantly the tumorigenicity of both circHER2 and its encoded 103 amino acids polypeptide (HER2-103aa) expressing cells in vivo [212].

### Therapeutic applications based on circRNA overexpression

CircRNA expression can be increased by direct delivery into cells. This approach is particularly effective in antagonizing oncogenic miRNAs by exploiting the property of circRNAs to act as ceRNAs/miRNA sponges. To obviate possible degradation effects, more efficient systems have adopted cassettes cloned inside lentivirus or adeno-associated virus vectors [213]. However, these vectors may lead to unforeseen adverse effects by producing simultaneously a substantial number of unnecessary cognate mRNA molecules. Alternative delivery of circRNA expression cassettes can be achieved through encapsulation within lipid and/or polymer nanoparticles. To illustrate the latter, a group of researchers ectopically overexpressed circFoxo3 by using plasmid-PEG- AuNPs target to cancer cells and demonstrated that circFoxo3 triggers stress- induced apoptosis, inhibition of tumor xenograft growth in vivo and increases overall survival [117].

### Therapeutic strategies based on circRNAs in response to cancer treatment failure

Gain- or loss-of-function-based therapeutic strategies targeting crucial circRNAs associated with cancer therapy resistance have also been investigated to overcome treatment failure. CircRNAs, such as CircRNA-SORE, have been shown to interfere with the activity of tyrosine kinase inhibitors, such as sorafenib. Upregulation of circRNA-SORE in sorafenib-resistant HCC cells ensures the cytoplasmic retention of the oncogenic protein YBX1 and prevents its ubiquitination and degradation by PRP19 in the nucleus and thereby favors resistance to sorafenib. Moreover, circRNA-SORE is loaded into exosomes that are trafficked from sorafenib-resistant cancer cells to sensitive cancer cells, and this helps to spread the resistance phenotype. By treating mice bearing subcutaneous sorafenib-resistant patient-derived xenografts with siRNAs targeting circRNA-SORE, researchers have demonstrated that the responsiveness to sorafenib treatment can be restored [214]. In a similar vein, the 174 amino acids long peptide (AKT3-174aa) encoded by circAKT3 has been shown to have important anti-tumorigenic roles and to be negatively involved in radiotherapy resistance. Mechanistically, AKT3-174aa competes with active phosphorylated PDK1, reduces AKT-thr308 phosphorylation, and acts as a negative regulator modulating PI3K/AKT signal intensity. This reduces cellular proliferation and antagonizes radiotherapy resistance. Downregulation of circAKT3 in glioblastoma causes the subsequent decrease in levels of its associated peptide and the development of a malignant glioblastoma phenotype. The injection of AKT3-174aa in mice models appears to restore glioblastoma cell sensitivity to radiotherapy [215]. Likewise, low levels of circ0025202 affecting the miR-182-5p/FOXO3a axis have been associated with the resistance of one of the most commonly used hormone therapy for hormone receptor (HR)-positive breast cancer patients (tamoxifen). In vivo experiments showed that overexpression of circ0025202 could shrink tumor growth and enhance tamoxifen efficacy [216]. Lastly, studies have revealed that circRNA17 can downregulate the expression of an androgen receptor splice variant 7 *via* sponging of one of its 3’ UTR targets, miR-181c-5p [217]. CircRNA17 overexpression in in vivo mouse model xenografted with enzalutamide-resistant cells, has shown to restore sensitivity to enzalutamide, used for prostate cancer treatment [217].

As discussed earlier, circUHRF1 plays a crucial role in sustaining anti-PD-1 resistance in HCC [218]. Using an in vivo xenograft model researchers have shown that the therapeutic suppression of circUHRF1 *via* shRNAs modulates the response to anti-PD-1 treatment and improves overall survival. This may prove an effective method of reversing resistance for ICIs by acting both on tumor cells and on associated dysfunctional immune microenvironment [218]. Immuno-suppression and development of anti-PD-1 therapy resistance in HCC have also been associated with circMET *via* the miR-30-5p/snail/DPP-4 axis. Treatment with sitagliptin, a dipeptidyl peptidase-4 (DPP-4) inhibitor generally used to treat type 2 diabetes, seems to obviate the resistant effects induced by the circMET. A combination of both sitagliptin and anti-PD-1 molecules has been shown to improve antitumor immunity in immunocompetent mice and is likely to be more effective in treating patients with HCC [219].

Further research is needed before specific circRNAs can be effectively exploited as clinically useful biomarkers or as therapeutic targets for cancer treatment. On-going studies are currently assessing the potential therapeutic benefits for cancer patients by bringing circRNA biology into clinical practice. Table 3 provides a snapshot of the range of projects currently in progress.

**Table 3:** Ongoing clinical trials on cancer-associated circRNAs as biomarkers or therapeutic targets (https://clinicaltrials.gov/).

Study title	Condition or disease	Intervention/treatment	Country of the study	Status of the study
*CIRcular and Non-coding RNAs as Clinically USeful Biomarkers in Pancreaticobiliary Cancers (CIRCUS) (NCT04584996)*	• Pancreatic Cancer • Biliary Tract Cancer		United Kingdom	Recruiting
*A Study of Blood Based Biomarkers for Pancreas Adenocarcinoma (NCT03334708)*	• Pancreatic Cancer • Pancreatic Diseases • Pancreatitis • Pancreatic Cyst	• Diagnostic Test: Blood Draw • Diagnostic Test: Tumor Tissue Collection • Diagnostic Test: Cyst Fluid	United States (5 hospitals involved)	Recruiting
*Thyroid Nodule Gene Sequencing in a Danish Population (NCT05377736)*	• Thyroid Nodule • (Diagnosis) Thyroid Cancer	• Genetic: Molecular analyses	Denmark	Enrolling by invitation
*Rediscovering Biomarkers for the Diagnosis and Early Treatment Response in NEN (REBORN) (NCT04464122)*	• Neuroendocrine Tumors • Neuroendocrine Neoplasm • Neuroendocrine Tumor Grade 1 • Neuroendocrine Tumor Grade 2 • Neuroendocrine Carcinoma	• Drug: Somatostatin analog; Chemotherapy	Italy	Recruiting
*Research on Precise Immune Prevention and Treatment of Glioma Based on Multi-omics Sequencing Data (NCT04792437)*	• Transcriptomics • Radiomics • Glioma	• Procedure: Surgery	China	Recruiting
*Gene Therapy and Combination Chemotherapy in Treating Patients With AIDS-Related Non-Hodgkin Lymphoma (NCT02337985)*	• AIDS-Related Burkitt Lymphoma • AIDS-Related Diffuse Large B-cell Lymphoma • AIDS-Related Plasmablastic Lymphoma • AIDS-Related Primary Effusion Lymphoma • HIV Infection • AIDS-Related Non-Hodgkin Lymphoma	• Drug: Prednisone • Biological: Rituximab • Drug: Etoposide • Drug: Doxorubicin Hydrochloride • Drug: Vincristine Sulfate • Drug: Cyclophosphamide • Biological: Filgrastim • Biological: Lentivirus Vector rHIV7-shI-TAR-CCR5RZ-transduced Hematopoietic Stem/Progenitor Cells	United States	Active, not recruiting

## Concluding remarks, perspectives and a note of caution

As sequencing approaches improve in terms of depth, accuracy, and read length [220], the annotation of new uncharacterized circRNAs, especially low abundance ones, continues to grow. Beyond their detection, a careful investigation to distinguish what is a splicing artifact from a functional circRNA remains essential. Appropriate controls and accurate validation using multiple approaches remain crucial to rule out false positive circRNAs. To tackle these challenges a series of guidelines in the field of circRNAs research have recently been published [221].

Many open questions regarding their biogenesis and biological functions, remain unanswered. It remains unclear whether or not they are exclusively co- or post-transcriptionally generated, how they are ultimately degraded, to what extent their structures might confer functional differences compared to their linear RNA counterparts and what proportion are translated into proteins. Moreover, many functional aspects of circRNAs might have not been uncovered yet due to the limitations of current approaches.

From a clinical perspective, the advent of circRNA research has opened up new exciting possibilities in cancer research, for better diagnosis and novel therapies. CircRNAs have been associated with a variety of physiological conditions and cell biology features including stemness and pluripotency. It has also been demonstrated that they have potential utility as valid biomarkers for early-cancer detection, diagnosis, prognosis and even prediction of response to therapies. However, how they impact cancer diagnosis and prevention remains debatable and sometimes even contradictory. CircRNAs often participate in more than one molecular mechanism in many tissues and diseases; targeting circRNAs therapeutically might lead to off-target effects in non-cancerous cells and tissue, making their clinical translation particularly challenging. Moreover, the discovery of multiple miRNAs sponged by the same circRNAs in different cancer types suggests that their contribution to a specific cancer phenotype is likely to be context-dependent. Additional related controversies surround the stoichiometry circRNA–miRNA–mRNA and the role of circRNAs as competitive endogenous RNAs [222, 223]. This is poorly understood in physiological conditions suggesting that the number of circRNAs able to effectively contribute to tumorigenesis by sponging miRNAs could be far lower than initially proposed. Measuring correct copy numbers and circRNA/miRNA ratio in cells and tissues remains fundamental in studying circRNAs and a necessary step required to understand how they function in normal development and disease.

The integration of artificial intelligence (AI) and circRNA transcriptome analysis holds great promise for advancing our understanding of this complex biological system, undoubtedly facilitating diagnostics and therapeutics in various pathological contexts including cancer. New approaches based on AI might help to identify shared and unique characteristics in features of circRNAs, such as their expression levels, alternative splicing patterns, co-expression networks, or specific structural motifs, all in different conditions. However, the success of using circRNA transcriptome data combined with AI for building classifiers will rely on high-quality data generation, careful experimental design, and rigorous computational analysis. The interpretability of AI models in this context remains challenging, as circRNA properties and functions are still not fully elucidated.

In summary, there is undoubtedly tremendous potential for circRNAs to have a significant impact on cancer diagnosis and treatment. The initial studies of circRNAs have necessarily focused on their discovery and characterization; what needs to follow in the near future is more detailed biological validation with patient samples in the context of clinical research. These experiments will need to be further corroborated with detailed functional studies in suitable model organisms. Once a more complete understanding of their functional biology is obtained, it will be possible to exploit the full potential of circRNAs in the clinic.

## Data Availability

All data are included in the published review manuscript.
